# Self-Propagating Combustion Synthesis, Luminescent Properties and Photocatalytic Activities of Pure Ca_12_Al_14_O_33_: Tb^3+^(Sm^3+^)

**DOI:** 10.3389/fchem.2018.00069

**Published:** 2018-03-20

**Authors:** Rong Liu, Yongsheng Yan, Changchang Ma

**Affiliations:** ^1^School of Chemistry, BaiCheng Normal University, Baicheng, China; ^2^Institute of Green Chemistry and Chemical Technology, Jiangsu University, Zhenjiang, China

**Keywords:** SPCS, Ca_12_Al_14_O_33_: Tb^3+^(Sm^3+^), pure phase, luminescent property, photocatalytic activity

## Abstract

The dual-functional Ca_12_Al_14_O_33_: Tb^3+^ and Ca_12_Al_14_O_33_: Sm^3+^ materials were prepared by the Self-Propagating Combustion Synthesis (SPCS) technology. The structure, morphology and light absorption property were investigated by XRD, FT-IR, UV-Vis DRS and SEM etc. The doping of Tb^3+^ and Sm^3+^ ions had not changed cubic structure of Ca_12_Al_14_O_33_ but leaded to the slight lattice dilatation and the red-shifts of absorption peaks/edges. The excitation and emission spectra indicated that Ca_12_Al_14_O_33_: Tb^3+^ and Ca_12_Al_14_O_33_: Sm^3+^ are superior green and red luminescent materials, respectively, and it displayed the distinctly refined structure characteristics which had importantly reference value for the energy level investigation of Tb^3+^ and Sm^3+^ ions. Meanwhile, Ca_12_Al_14_O_33_: Tb^3+^ and Ca_12_Al_14_O_33_: Sm^3+^ also exhibited the improved photocatalytic degradation for removing dye MB compared with bare Ca_12_Al_14_O_33_.

## Introduction

In recent years, the widespread application of rare-earth luminescent materials (RELMs) has been proved to promote the upgrading of products in display area (Yang et al., 2001; Xie et al., 2002; Zhang et al., 2017). RELMs have been a kind of essential materials in energy-efficient lighting and electronic information industry owing to their low-cost, good color display, pollution-free, long-life, nontoxic advantages and so on (Li et al., 2009; Yu et al., 2009). In addition, RELMs are widely used in agriculture, environmental sanitation, medical care, simulate natural light source, etc. special application fields (Li and Lin, 2010; Gai et al., 2014; Escudero et al., 2016, 2017).

It is always the intensive research subject to explore the novel oxide and composite oxide RELMs with high luminescent efficiency and favorable thermal stability. Especially, the RELMs based on alkaline-earth metal aluminates composite oxides have become the research focus due to their unique advantages of high luminescent efficiency, stable chemical properties, high quenching temperature, corrosion resistance, low-cost and nontoxic, pollution-free characteristics (Feng et al., 2010; Yu et al., 2013; Min et al., 2014). For example, high-efficiency green MgAl_11_O_19_: Ce^3+^, Tb^3+^ (Jung et al., 2005) and blue BaMgAl_10_O_17_: Eu^2+^ (Kim et al., 2002) luminescent powders used Mg(Ba)O-Al_2_O_3_ as hosts had widely been applied in the world. Also, there are many reports on the blue-luminescent materials using SrO-Al_2_O_3_ system as hosts, including Sr_2_Al_6_O_11_ (Takeda et al., 2002), Sr_4_Al_14_O_25_ (Garcia et al., 2016), SrAl_2_O_4_ (Sohn et al., 2002), and SrAl_4_O_7_ (Singh et al., 2016), etc., as well as red-luminescent materials using LiAlO_2_ (Lee et al., 2012), LiAl_5_O_8_ (Singh and Rao, 2008), and CaAl_12_O_19_ (Brik et al., 2011) as hosts. However, many of above materials are prepared by the traditional solid phase calcined method, which has the obvious deficiency of energy consumption because of high synthesis temperature. Especially for calcium aluminate host materials, they are difficult to obtain pure phase product owing to generation of many phases together in the preparation. Existence of mixed phases may influence on luminescence performance of RELMs when they are used as host materials. Therefore, in this paper, two pure phase RELMs using Ca_12_Al_14_O_33_ as host material and Tb^3+^(Sm^3+^) as active ions are successful prepared by a simple SPCS technique. The synthesis temperature is significantly reduced. It is worth noting that both of Ca_12_Al_14_O_33_: Tb^3+^ and Ca_12_Al_14_O_33_: Sm^3+^ exhibit dual-functional features, not only show outstanding luminescent properties but also disply superior photocatalytic activities, which may have potential application prospects in display and catalysis fields.

## Experimental

Al(NO_3_)_3_·9H_2_O, Ca(NO_3_)_2_·4H_2_O, urea and concentrated nitric acid are analytical reagent. The purity of Tb_4_O_7_ and Sm_2_O_3_ were ≥99.9%. The reaction materials were weighted making use of electronic balance in accordance with Ca_12−x_Al_14_O_33_: xTb^3+^(Sm^3+^) (*x* = 0.01–0.05) stoichiometric ratio, respectively. The appropriate ratio Tb_4_O_7_ and Sm_2_O_3_ were transferred to 100 ml beakers and dissolved via a little concentrated HNO_3_ (A.R.), respectively. After evaporating to dryness, Al(NO_3_)_3_•9H_2_O, Ca(NO_3_)_2_•4H_2_O, CO(NH_2_)_2_ and appropriate distilled water were added. Keeping on stirring, dissolving and heating until the solution was evaporated to be viscous, Subsequently, the beaker was put into a muffle furnace at 500°C. After a few minutes, the reaction material burned quickly and emited a bright flame. The entire combustion process was completed within 5–7 min. The white mushroom-shaped precursors with loose, porous and soft property were obtained. Finally, the precursors were grinded 30 min and transferred into the corundum crucible and calcined in the muffle furnace at 1,100°C for 6 h to obtain white products.

X-ray powder diffraction (XRD) patterns of products were recorded on Rigaku Dmax-2200 powder diffractometer (Cu K_α1_ = 1.54056 × 10^−10^ m, scanning speed 6° min^−1^, scanning 2θ range 3–80° with steps of 0.02°). Luminescent spectra were measured via F4500 fluorescence spectrophotometer using Xe lamp as the excitation source (EX slit 2.5 nm/EM slit 2.5 nm, scanning speed 12,000 nm min^−1^). Morphologies were observed with S-3000N scanning electron microscopy (SEM). All the measurements were carried out at room temperature. FT-IR absorption spectra were measured on FT-IR360 infrared spectrometer using KBr pellets in the region of 4,000–400 cm^−1^. The UV-vis diffuse reflectance spectra (DRS) of the samples were recorded on a UV–vis spectrophotometer (PG, TU-1900) with BaSO_4_ as the background at room temperature.

The dye methylene blue (MB) solution (10 mg L^−1^, 100 ml) containing 0.1 g sample was irradiated under the UV-Visible light with a 300 W Xe arc lamp. Before the irradiation, it was stirred for 30 min in the dark to achieve the adsorption-desorption equilibrium between dye MB and sample. The absorbance of dye MB solution was monitored by UV-vis spectrophotometer (PG, TU-1901) every 5 min.

## Results and discussion

### Structure analysis of the as-prepared samples

**Figure 1:**
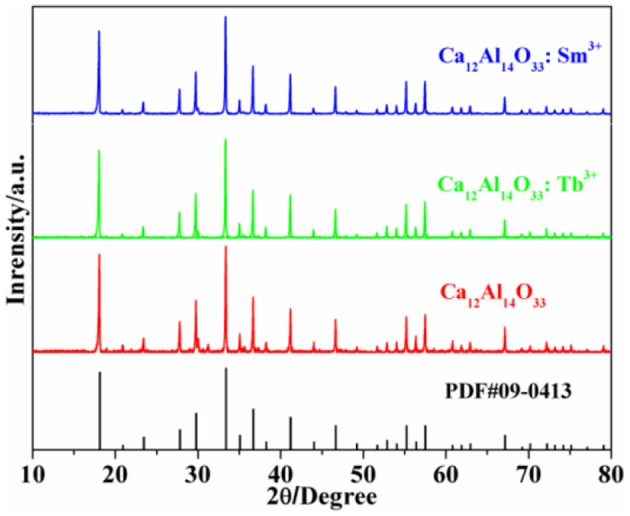
XRD patterns of the as-prepared samples.

Figure 1 shows the X-ray powder diffraction (XRD) patterns of Ca_12_Al_14_O_33_, Ca_12_Al_14_O_33_: Tb^3+^ and Ca_12_Al_14_O_33_: Sm^3+^. All diffraction patterns accord with JCPDS PDF#09-0413 cards well. No other miscellaneous diffraction peaks are observed, which proves that the three samples are completely transformed into Ca_12_Al_14_O_33_ crystalline phase without generating other types of calcium aluminates. Meanwhile, the sharp and intense diffraction peaks indicate that the as-prepared samples have high crystalline. We used PowderX (Dong, 1999) to execute smooth, deduct back bottom, and isolate K_α2_ line diffraction peak of Cu target, seek peak and perform the index treatment of each diffraction peak for the obtained XRD patterns. The results demonstrate that crystal cells of Ca_12_Al_14_O_33_: Tb^3+^ and Ca_12_Al_14_O_33_: Sm^3+^ belong to the cubic crystal system with an *I*-43d space group, and the crystal cell parameters are *a* = 11.9895 Å and *a* = 11.9892 Å, *Z* = 2, respectively. The crystal cell parameters of two samples are slightly bigger than that of Ca_12_Al_14_O_33_ (11.9820 Å), which means that lattice mild expansion takes place after a small amount of Tb^3+^ or Sm^3+^ ions entering the crystal lattice to replace Ca^2+^ ions in Ca_12_Al_14_O_33_.

### FT-IR absorption spectra of the as-prepared samples

**Figure 2:**
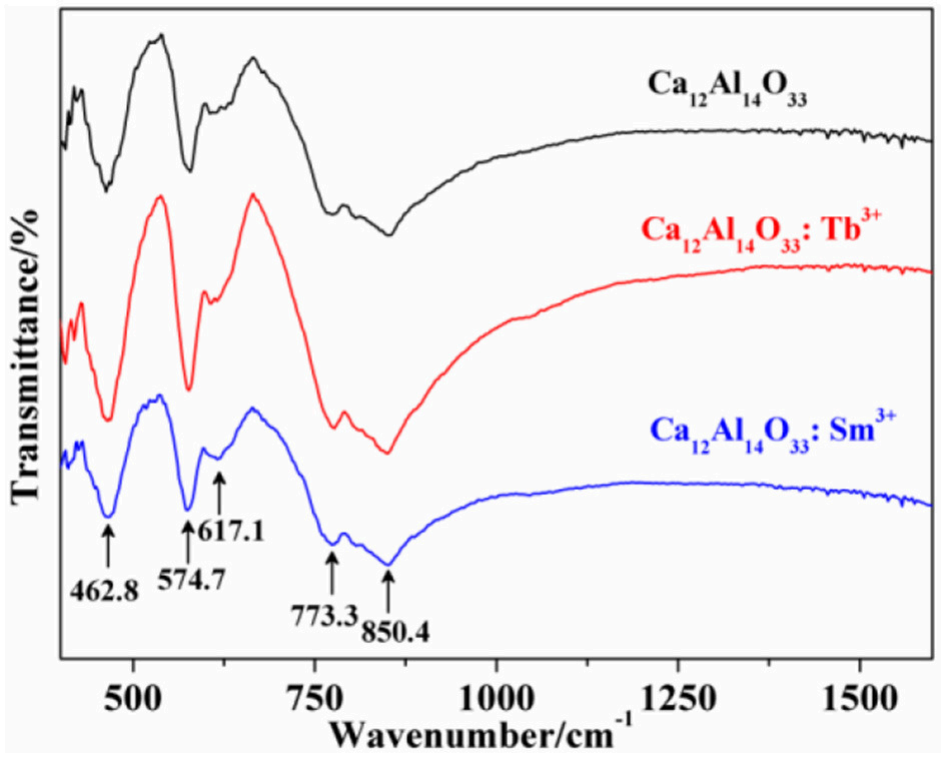
FT-IR absorption spectra of the as-prepared samples.

The fourier transforming infrared (FT-IR) absorption spectra of Ca_12_Al_14_O_33_, Ca_12_Al_14_O_33_: Tb^3+^ and Ca_12_Al_14_O_33_: Sm^3+^ are shown in Figure 2 which are basically coincide with the results of the reported Ca_12_Al_14_O_33_ (Tas, 1998). The absorption band of condensate and isolation AlO_4_ locates at the range of 900–700 cm^−1^ and 800–650 cm^−1^, as well as the absorption band of condensate and isolation of AlO_6_ locates at the range of 680–500 and 530–400 cm^−1^, respectively (Yi et al., 2015). As a consequence, the strong broad band absorptions at around 800 cm^−1^ in Figure 2 are attributed to AlO_4_ stretching vibration, which are composed by two absorption peaks at 850.40 and 773.4 cm^−1^. Those results demonstrate there are two AlO_4_ tetrahedral structures in the lattice, which is accordance with the obtained structure in the Ca_12_Al_14_O_33_ unit cell (Boysen et al., 2007). Because AlO_6_ octahedral structure is inexistence in the Ca_12_Al_14_O_33_ unit cell and the absorption band located at 400–620 cm^−1^ shows two group strong peaks in Ca_3_Al_2_O_6_, Ca_12_Al_14_O_33_, CaAl_12_O_19_, CaAl_4_O_7_, CaAl_2_O_4_, etc. calcium aluminate, the peaks located 617.1, 574.71, and 462.8 cm^−1^ should derive from characteristic vibration absorption of Al-O bonds. All the above prove that Ca_12_Al_14_O_33_ crystal lattice structure is no more obviously changed except only slight distortion when the Ca^2+^ ions are replaced by Tb^3+^ or Sm^3+^, which is consistent with XRD analysis results.

### UV–vis DRS of the as-prepared samples

**Figure 3:**
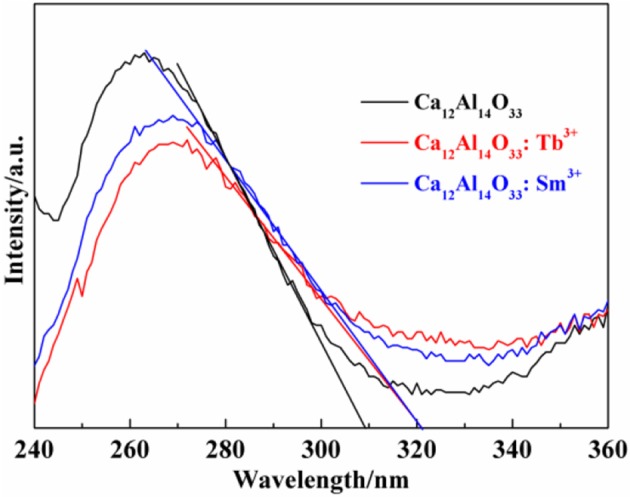
UV-Vis DRS spectra of the as-prepared samples.

The light absorption ability of the as-prepared samples is evaluated by the UV-vis diffuse reflectance spectra (DRS). As shown in Figure 3 all samples exhibit strong ultraviolet light absorption characteristics located at 240–320 nm. The steep shapes indicate that the intense absorptions are not due to the transition from the impurity level but band-gap transition (Li et al., 2015, 2017). It is noted that the absorption peak and absorption edge of pure Ca_12_Al_14_O_33_ are located at 263 nm and 309 nm, respectively. However, after doping Tb^3+^ and Sm^3+^ ions, the absorption peaks and absorption edges of Ca_12_Al_14_O_33_: Tb^3+^ and Ca_12_Al_14_O_33_: Sm^3+^ have apparent red-shift compared with that of original Ca_12_Al_14_O_33_ sample, which is red-shift of about 6 and 12 nm toward the longer wavelengths and located at 269 and 321 nm, respectively. The optical absorption change may result from the doping effect of Tb^3+^and Sm^3+^ causing slight lattice expansion of Ca_12_Al_14_O_33_.

### SEM images of the as-prepared samples

**Figure 4:**
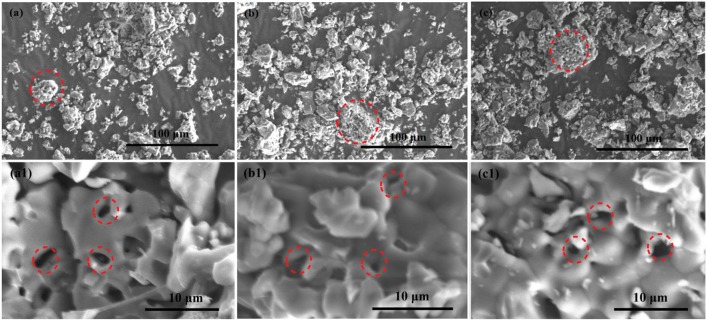
SEM images of Ca_12_Al_14_O_33_ **(a,a1)**, Ca_12_Al_14_O_33_: Tb^3+^ **(b,b1)**, and Ca_12_Al_14_O_33_: Sm^3+^ **(c,c1)** samples.

The morphologies of the as-prepared samples are observed by photomicrographs measured via scanning electron microscopy (SEM). As shown in Figures 4a–c the images with low magnification of Ca_12_Al_14_O_33_, Ca_12_Al_14_O_33_: Tb^3+^ and Ca_12_Al_14_O_33_: Sm^3+^ present porous and irregular bulk feature, which have the obvious agglomeration gathered by some particles. It may result from high-temperature calcination for a long time. Correspondingly, Figures 4a1–c1 are the high-magnification SEM images of Ca_12_Al_14_O_33_, Ca_12_Al_14_O_33_: Tb^3+^ and Ca_12_Al_14_O_33_: Sm^3+^, respectively. Those samples exhibit honeycomb distribution composed of crystalline granular adhesions with few microns, smooth surface and better crystallization effect. This result can be attributed to the following reasons: in the SPCS process, a lot of gases are released to damage the formation of massive structures owing to urea burning, so that crystal nuclei growth is along to direction to formation of sphere shapes containing the lower surface energy. The shapes of as-prepared samples influence the luminescence performance to some extent and lots of researches have shown spherical surface are conducive to enhancing luminescent intensity (Kang et al., 2000).

### Luminescent properties of the as-prepared samples

**Figure 5:**
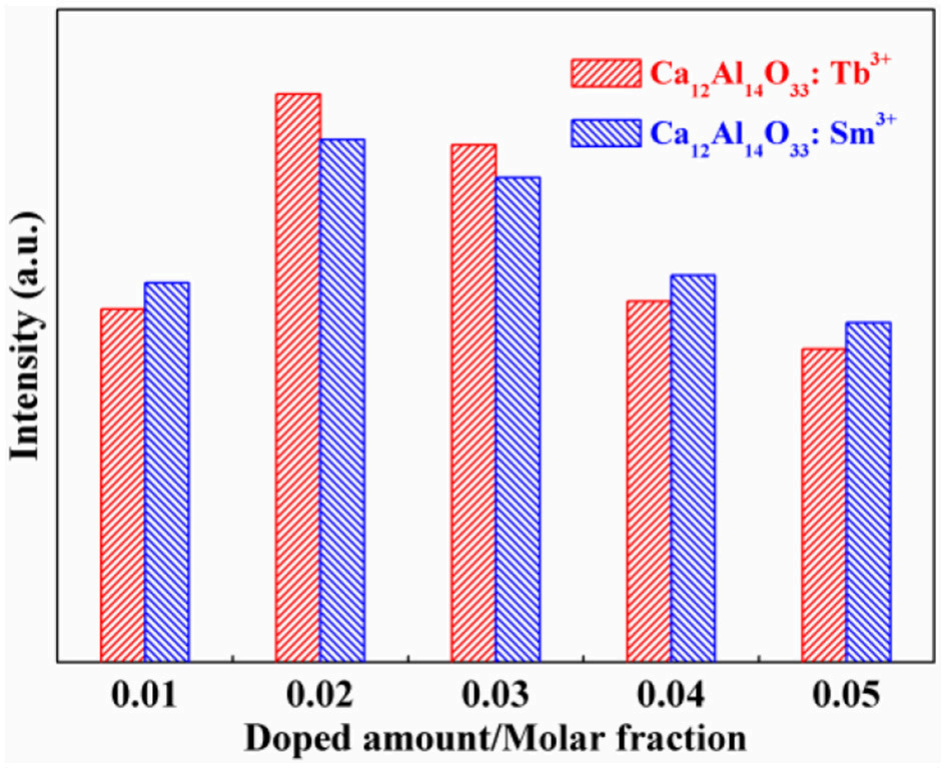
Effect of doped amount on the luminescent intensity of the as-prepared samples.

The bare Ca_12_Al_14_O_33_ has no luminescence property without doping earth ions. However, when Ca_12_Al_14_O_33_ is doped by Tb^3+^ and Sm^3+^ ions, it will produce characteristic luminescent emission of these two ions. Figure 5 is the luminescent emission intensity of Ca_12_Al_14_O_33_: Tb^3+^ and Ca_12_Al_14_O_33_: Sm^3+^ with different doping amount, where the standard of comparison is evaluated by the strongest energy level transition of ^5^D_4_→ ^7^F_5_ for Tb^3+^ and ^4^G_5/2_→ ^6^H_7/2_ for Sm^3+^ ions. Obviously, the luminescent intensity of Ca_12_Al_14_O_33_: Tb^3+^ and Ca_12_Al_14_O_33_: Sm^3+^ present increase first and then decrease with increasing the doping amount of Tb^3+^ and Sm^3+^ ions. When the doping amount of Tb^3+^ and Sm^3+^ is 0.02 of molar fraction, the luminescent emissions reach up the strongest intensity, because the excess rare earth ions usually produce fluorescence quenching effect that results in reduction of luminescent emission intensity.

**Figure 6:**
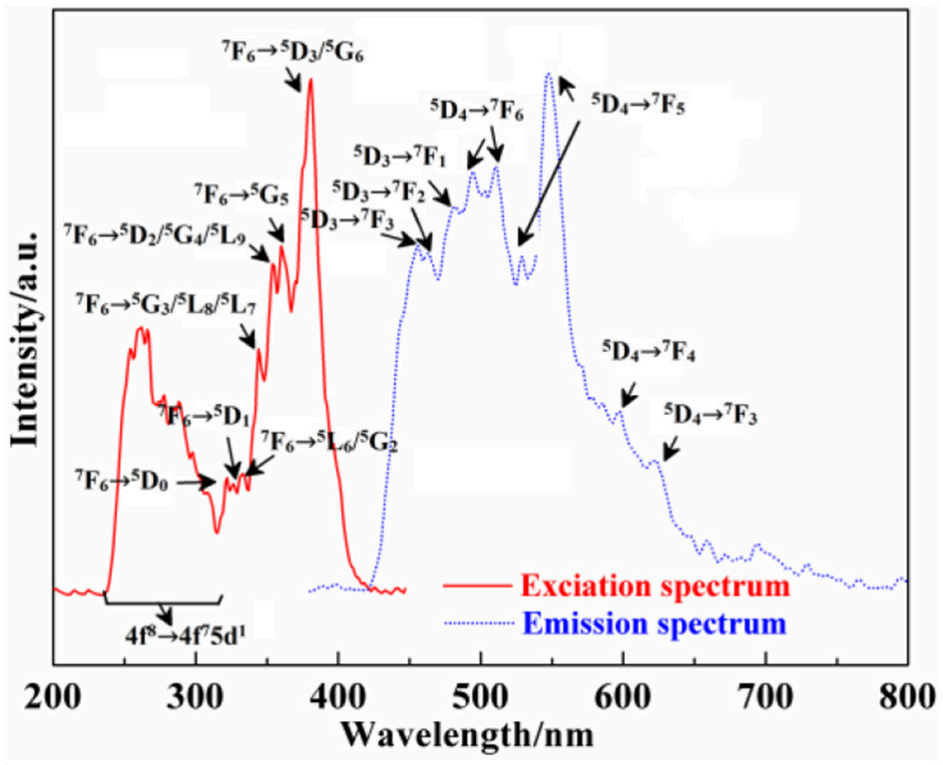
Excitation and emission spectra of Ca_12_Al_14_O_33_: Tb^3+^.

Figure 6 shows the excitation (λ_em_ = 545 nm) and emission spectra (λ_ex_ = 359 nm) of Ca_12_Al_14_O_33_: Tb^3+^. Because the 4f^7^ state of Tb^3+^ ions has a stable semi-filled electron configuration, Tb^3+^ ions can be excited by the relative low energy, whose excitation band is always composed by f → f and f → d transition. Therefore, from the excitation spectrum of Ca_12_Al_14_O_33_: Tb^3+^, we can draw to a conclusion that the excitation band at the short-wave 220–315 nm corresponds to the 4f^8^ → 4f^7^5d^1^ transition absorption of Tb^3+^ ions, and the excitation band located at 320–400 nm originates from f → f transition absorption, where the energy level transition of different absorption peaks at 381, 360, 354, 344, 333, 326, and 322 nm may be attributable to the energy level transition absorption of ^7^F_6_ → (^5^D_3_/^5^G_6_), ^5^G_5_, (^5^D_2_/^5^G_4_/^5^L_9_), (^5^G_3_/^5^L_8_/^5^L_7_), (^7^F_6_→ ^5^L_6_/^5^G_2_), ^5^D_1_, ^5^D_0_, respectively (Fu et al., 2010). Furthermore, from the emission spectrum of Ca_12_Al_14_O_33_: Tb^3+^, we can find that the common linear emission peak of Tb^3+^ ion presents wide band distribution, which is different from other common fluorescent materials (Fu et al., 2010; Dong, 2011). The emission peaks located at (495, 511), (529, 547), 597, and 623 nm come from the energy level transition of ^5^D_4_→ ^7^F_6_, ^7^F_5_, ^7^F_4_, ^7^F_3_, respectively (Fu et al., 2010). Due to the large *J*-value of the transition, the crystal field will result in the splitting of these energy levels. Meanwhile, to eliminate the parity-forbidden transition of Tb^3+^ ions, the opposite parity energy level of 4f configuration is not the charge transfer band, but is the 4f^7^5d^1^ energy level with low energy. ^5^D_4_→ ^7^F_6_ electric dipole transition of Tb^3+^ ions is not as sensitive to ligand environment as the ^5^D_0_→ ^7^F_2_ electric dipole transition of Eu^3+^ ions. Therefore, ^5^D_4_→ ^7^F_5_ magnetic dipole transition is the strongest in the emission spectra, so that the Ca_12_Al_14_O_33_: Tb^3+^ sample emits green light when it is excited under the ultraviolet light. Meanwhile, the stronger emission peaks located at 456, 463, and 482 nm origin from the higher excited states ^5^D_3_→ ^7^F_3_, ^7^F_2_, ^7^F_1_ energy level transitions, respectively, which indicates that there is lightly cross relaxation between ^5^D_3_ and ^5^D_4_ energy levels (Fu et al., 2010; Dong, 2011).

**Figure 7:**
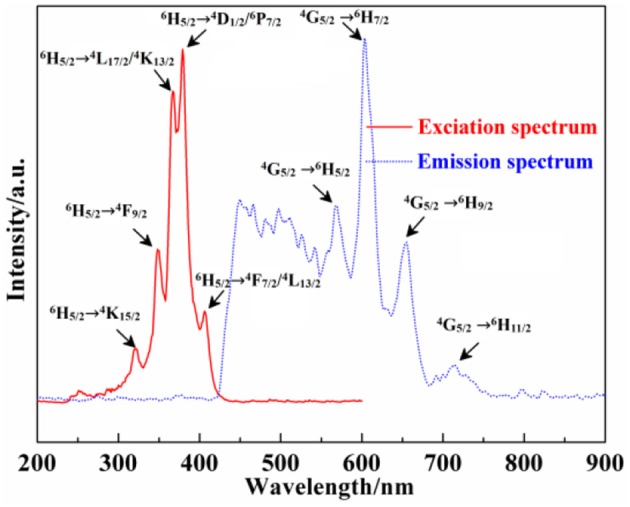
Excitation and emission spectra of Ca_12_Al_14_O_33_: Sm^3+^.

Moreover, the excitation (λ_em_ = 604 nm) and emission spectra (λ_ex_ = 382 nm) of Ca_12_Al_14_O_33_: Sm^3+^ are exhibited in Figure 7 As can be seen from the excitation spectrum, there are five groups of excitation peaks in the range from 320 to 420 nm. It corresponds to the high energy f → f configuration transition absorption of Sm^3+^ ions, where the excitation peaks located at 406, 379, 372, 367, and 348 nm may be belong to the transition absorption of ^6^H_5/2_ → (^4^F_7/2_/^4^L_13/2_),(^4^D_1/2_/^6^P_7/2_),(^6^H_5/2_→ ^4^L_17/2_/^4^K_13/2_),^4^F_9/2_,^4^K_15/2_, respectively (Zhang et al., 2010). From the emission spectrum, the emission peaks located at 568 nm, 604 nm, 655 nm and 714 nm come from the energy level transition of ^4^G_5/2_→ ^6^H_5/2_, ^6^H_7/2_, ^6^H_9/2_, ^6^H_11/2_, respectively (Zhang et al., 2010). P. S. May and coworkers (May et al., 1992) found that, ^4^G_5/2_→ ^6^H_5/2_ mainly belongs to a magnetic dipole transition, and partly belongs to electric dipole transition; though ^4^G_5/2_→ ^6^H_7/2_ is magnetic dipole transition, the electric dipole transition plays a predominance function; ^4^G_5/2_→ ^6^H_9/2_ is assigned to electric dipole transition, but the magnetic dipole transition is forbidden. In addition, according to the results reported by Tamura (Tamura and Shibukawa, 1993), if Sm^3+^ ion mainly occupies the asymmetry center, it can produce typical emission near 650 nm. On the contrary, if Sm^3+^ ion mainly occupies the symmetry center, it can produce typical emission near 602 nm. Therefore, as can be seen from the emission spectrum of Ca_12_Al_14_O_33_: Sm^3+^, the transition emission intensity of ^4^G_5/2_→ ^6^H_7/2_ is bigger than that of ^4^G_5/2_→ ^6^H_9/2_, and the transition emission intensity of ^4^G_5/2_→ ^6^H_5/2_ is bigger than that of ^4^G_5/2_→ ^6^H_9/2_, which indicate the Sm^3+^ ions mainly occupies the symmetry center in the lattice. Besides, a strong and refined-structure transition emission peak is observed at 450–580 nm, which may come from the transition emission of high energy level of Sm^3+^ ions.

### Photocatalytic activities of the as-prepared samples

**Figure 8:**
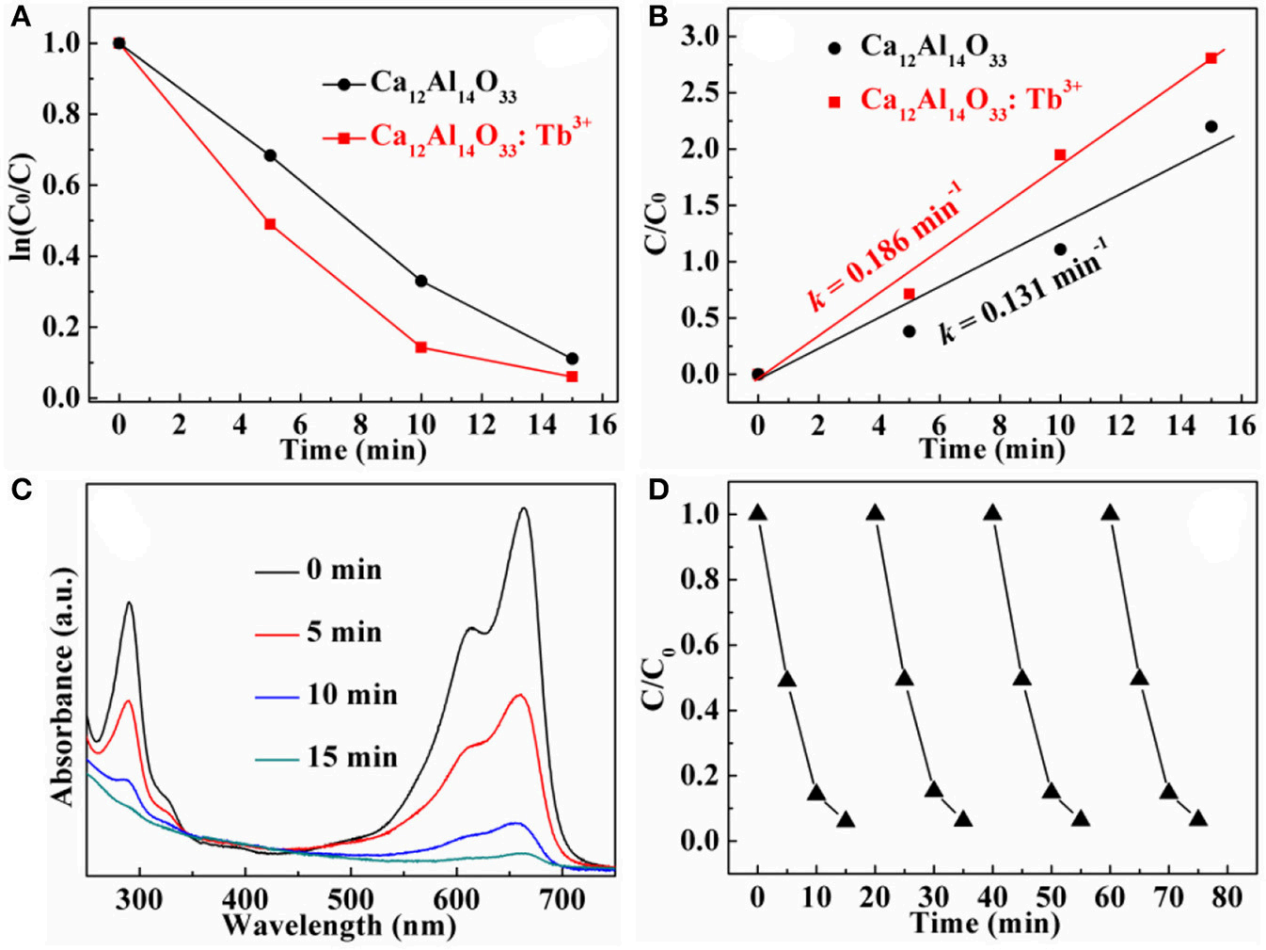
Dynamic curves **(A)** and plots of ln(c_0_/c) vs. time **(B)** of dye MB solution over Ca_12_Al_14_O_33_ and Ca_12_Al_14_O_33_: Tb^3+^, absorbance variations **(C)** and cycle degradation runs **(D)** of dye MB solution over Ca_12_Al_14_O_33_: Tb^3+^.

**Figure 9:**
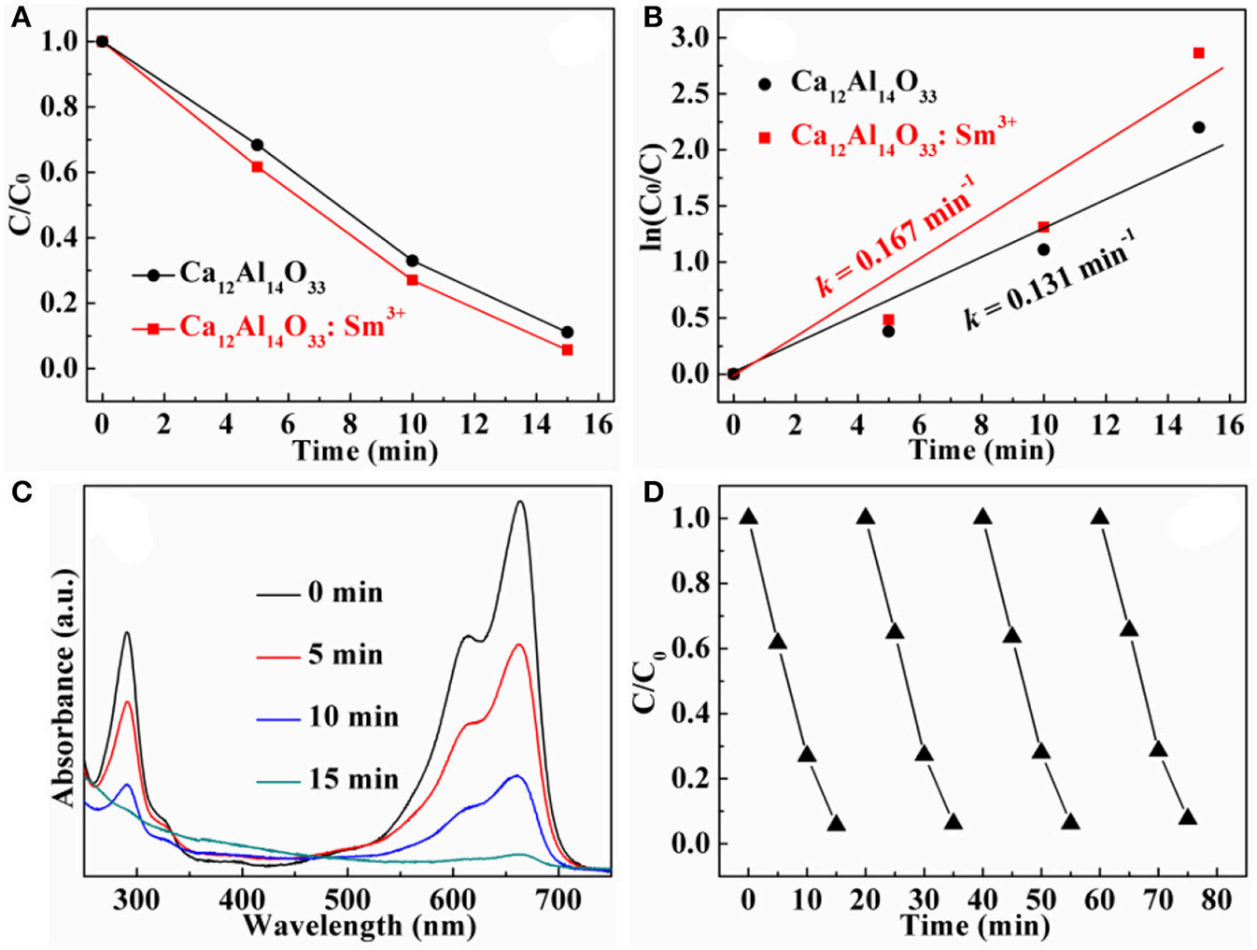
Dynamic curves **(A)** and plots of ln(c_0_/c) vs. time **(B)** of dye MB solution over Ca_12_Al_14_O_33_ and Ca_12_Al_14_O_33_: Sm^3+^, absorbance variations **(C)** and cycle degradation runs **(D)** of dye MB solution over Ca_12_Al_14_O_33_: Sm^3+^.

The dye methylene blue (MB) is typical organic pollutant, which is usually used as target molecule to evaluate the photocatalytic ability of the photocatalytic materials (Dong et al., 2014c). Figures 8A, 9A show the degradation dynamic curves of dye MB over the Ca_12_Al_14_O_33_: Tb^3+^ and Ca_12_Al_14_O_33_: Sm^3+^ samples, respectively. After running 15 min, two samples all show the high degradation rates is more than 98% for removing dye MB, respectively, whose photocatalytic activities are obviously higher than that of bare Ca_12_Al_14_O_33_. Moreover, the kinetic curves of dye MB degradation can be approximated as the pseudo-first-order process (Dong et al., 2013, 2014a,b,c; Li et al., 2014, 2016). By plotting the ln(*c*_0_/*c*) vs. time and making linear fitting for dynamic curves in Figures 8B, 9B the removal rate constants (*k*) of dye MB are estimated to be 0.186 and 0.167 min^−1^, respectively, which is distinctly higher than that of bare Ca_12_Al_14_O_33_ (0.131 min^−1^). Moreover, according to the absorbance variations of dye MB solutions in Figures 8C, 9C in the photocatalytic reaction process, there are no shifting of the maximum absorption position of dye MB solution at 664 nm. In addition, the absorption peak at 293 nm in the ultraviolet range also vanishes, which implies that the benzene/heterocyclic rings of dye MB molecule may be completely decomposed, leading to the thorough mineralization of dye MB (Dong et al., 2013, 2014a,b,c; Li et al., 2014, 2016). Meanwhile, in order to investigate the reusability of the Ca_12_Al_14_O_33_: Tb^3+^ and Ca_12_Al_14_O_33_: Sm^3+^ samples, the circle degradation experiments of dye MB solution over the Ca_12_Al_14_O_33_: Tb^3+^ and Ca_12_Al_14_O_33_: Sm^3+^ samples are all performed. As shown in Figures 8D, 9D the experimental results indicate the photocatalytic ability of two samples does not show obviously loss after four recycles, indicating that they have superior stability and reusability.

### The possible luminescent and photocatalytic mechanism

**Figure 10:**
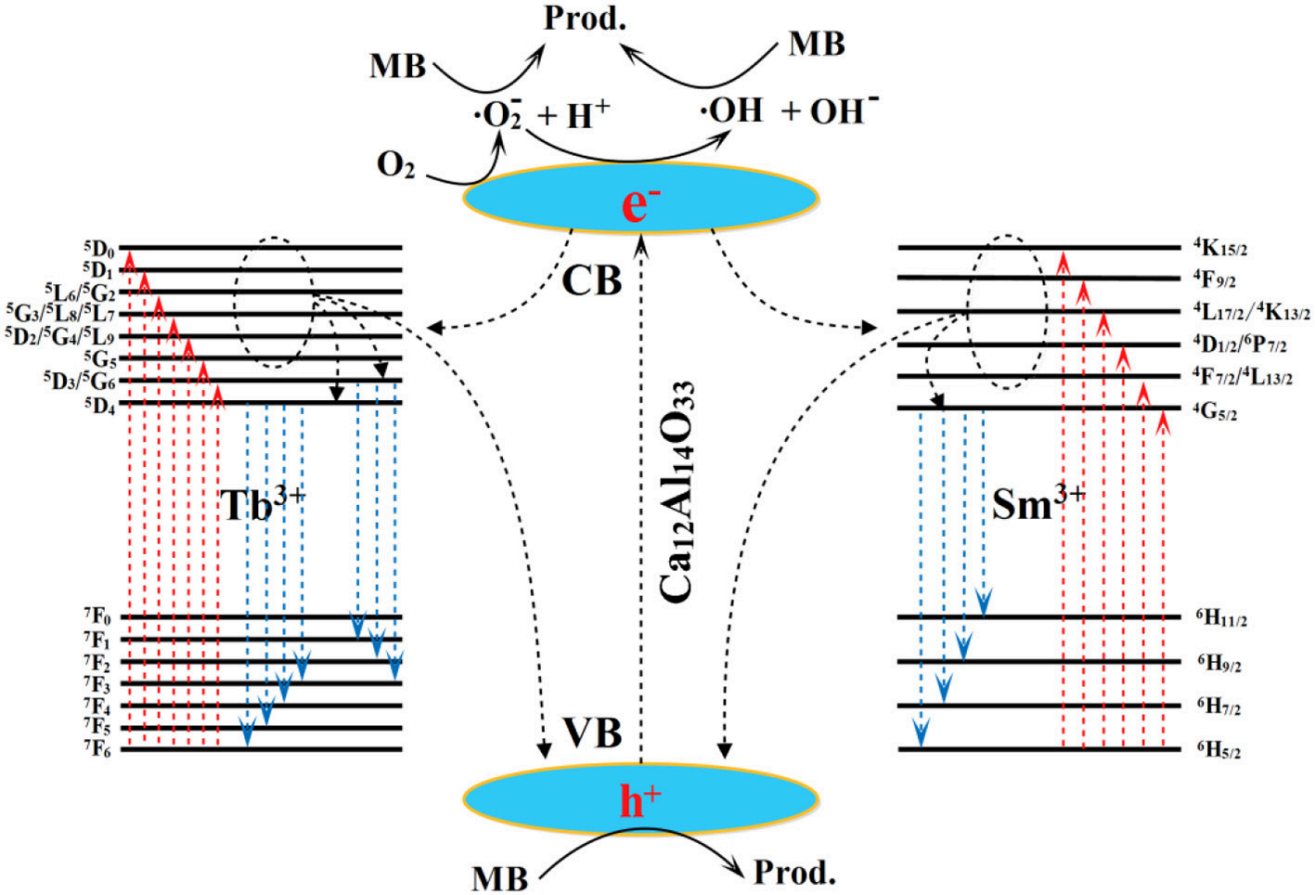
The possible transfer behavior of charge carriers, luminescent emission and photocatalytic mechanism.

The possible transfer behavior of charge carriers, luminescent emission and photocatalytic mechanism are shown in Figure 10 Under the UV-vis light excitation, the Ca_12_Al_14_O_33_ host and Tb^3+^ and Sm^3+^ ions are all excited at the same time. Electrons in the VB of Ca_12_Al_14_O_33_ host transfer into the corresponding CB, as well as electrons in the ground state ^7^F_6_ of Tb^3+^ and ^6^H_5/2_ of Sm^3+^ ions transfer into the (^5^D_3_/^5^G_6_), ^5^L_10_, ^5^G_5_, (^5^D_2_/^5^G_4_/^5^L_9_), (^5^G_3_/^5^L_8_/^5^L_7_), (^5^L_6_/^5^G_2_), ^5^D_1_, ^5^D_0_ states and (^4^F_7/2_/^4^L_13/2_), (^4^D_1/2_/^6^P_7/2_), (^4^L_17/2_/^4^K_13/2_), ^4^F_9/2_, ^4^K_15/2_ states of them, respectively. At the luminescent process, the electrons in the excitation states ^5^D_4_ and ^5^D_3_ return to ^7^F states of Tb^3+^ ions to generate luminescence, such as ^5^D_4_→ ^7^F_6_, ^7^F_5_, ^7^F_4_, ^7^F_3_ and ^5^D_3_→ ^7^F_3_, ^7^F_2_, ^7^F_1_ transition emission. It should be pointed out that parts of electrons in the CB of Ca_12_Al_14_O_33_ host and the high energy levels of Tb^3+^ can transfer into ^5^D_4_ and ^5^D_3_ states by means of multi-phonon assisted relaxation effect to enhance luminescent performance. Similarly, the electrons in the excitation state ^4^G_5/2_ return to ^6^H states of Sm^3+^ ions to generate luminescence, such as ^4^G_5/2_→ ^6^H_5/2_, ^6^H_7/2_, ^6^H_9/2_, ^6^H_11/2_ transition emission. The parts of electrons in the CB of Ca_12_Al_14_O_33_ host and the high energy levels of Sm^3+^ also can transfer into ^4^G_5/2_ state by means of multi-phonon assisted relaxation effect to enhance luminescent performance. In the photocatalytic degrading MB process, parts of the electrons in the CB of the calcium aluminate host migrate to Ca_12_Al_14_O_33_ host surface and are captured by O_2_ molecules in water to yield superoxide radicals (•${\text{O}}_{2}^{-}$). The superoxide radicals may react with H^+^ ions to further transform into hydroxyl radicals (•OH). Finally, the superoxide radicals, hydroxyl radicals and holes all decompose MB dye molecules. In MB degradation process, the optical absorption increase may result from slight lattice expansion of Ca_12_Al_14_O_33_ host owing to the doping effect of Tb^3+^ and Sm^3+^ ions, which may be the main reason for the improved photocatalytic performance.

## Conclusions

The dual-functional Ca_12_Al_14_O_33_: Tb^3+^ and Ca_12_Al_14_O_33_: Sm^3+^ materials with single phase and good crystallinity are prepared by the SPCS technology. The investigation results indicate that there are no changes of cubic crystal structure after introducing Tb^3+^ or Sm^3+^ ions into Ca_12_Al_14_O_33_ besides a slight lattice expansion. When the doping amount of Tb^3+^ and Sm^3+^ is 0.02 of molar fraction, both two samples show the maximum luminescent intensity. The excitation spectra of two samples are mainly from the f → f transition absorption, and Ca_12_Al_14_O_33_: Tb^3+^ sample also appears 4f^8^ → 4f^7^5d^1^ transition absorption at the short-wave region. In the emission spectra of two samples, the refined character emission can be observed, in which the transfer emissions of Tb^3+^ ions mainly come from ^5^D_4_→ ^7^F_6_, ^7^F_5_, ^7^F_4_, ^7^F_3_ and ^5^D_3_→ ^7^F_3_, ^7^F_2_, ^7^F_1_, as well as the transfer emissions of Sm^3+^ ions come from ^4^G_5/2_→ ^6^H_5/2_, ^6^H_7/2_, ^6^H_9/2_, ^6^H_11/2_ and high-energy transition emission at 450-580 nm, respectively. Meanwhile, two samples also exhibit the high photocatlytic degradation activity, stability and reusability for removing dye MB pollution. These two dual-functional materials may possess the potential application in the display device and dye wastewater treatment.

## Author contributions

RL is in charge of synthesis and characterization of materials, and writing manuscript. YY is in charge of designing experimental plan and revising manuscript. CM is in charge of the performance test of materials.

### Conflict of interest statement

The authors declare that the research was conducted in the absence of any commercial or financial relationships that could be construed as a potential conflict of interest.
